# Hypervirulence Markers Among Non-ST11 Strains of Carbapenem- and Multidrug-Resistant *Klebsiella pneumoniae* Isolated From Patients With Bloodstream Infections

**DOI:** 10.3389/fmicb.2020.01199

**Published:** 2020-06-18

**Authors:** Ping Shen, Björn Berglund, Yong Chen, Yanzi Zhou, Tingting Xiao, Yonghong Xiao, Kai Zhou

**Affiliations:** ^1^Collaborative Innovation Center for Diagnosis and Treatment of Infectious Diseases, State Key Laboratory for Diagnosis and Treatment of Infectious Diseases, The First Affiliated Hospital, College of Medicine, Zhejiang University, Hangzhou, China; ^2^Department of Biomedical and Clinical Sciences, Linköping University, Linköping, Sweden; ^3^Shenzhen Institute of Respiratory Diseases, Second Clinical Medical College (Shenzhen People’s Hospital), Jinan University; First Affiliated Hospital (Shenzhen People’s Hospital), Southern University of Science and Technology, Shenzhen, China

**Keywords:** bloodstream infection, multidrug resistance, carbapenem resistance, *Klebsiella pneumoniae*, hypervirulence

## Abstract

Multidrug-resistant *Klebsiella pneumoniae* and hypervirulent *K. pneumoniae* (hvKP) have traditionally been considered two individual populations; however, strains displaying both phenotypes have emerged during the recent decade. Understanding the genotypic and phenotypic basis of the convergence could be of clinical importance. In this study, we aimed to evaluate the pathogenicity associated with different combinations of genotypes (i.e., sequence types, virulence factors, and capsular serotypes) and phenotypes (i.e., hypermucoviscosity and drug susceptibility) in *K. pneumoniae.* A total of 11 *K. pneumoniae* isolates causing bloodstream infections were included in the study, and they were assigned to seven STs (ST23, ST15, ST268, ST660, ST86, ST65, and ST1660) and carried various K-loci (KL1, KL2, KL16, KL20, and KL24). Hypermucoviscosity was observed for six isolates. *bla*_KPC–2_ was detected in six carbapenem-resistant isolates, and the remaining ones were either multidrug-resistant or resistant to two types of antibiotics. Aerobactin- and yersiniabactin-encoding genes were detected in all isolates. Although *rmpA2* was detected in all isolates, most contained frameshift mutations (82%). Genes encoding salmochelin, RmpA, and PEG344 were detected in seven isolates. Colibactin-encoding genes were carried by six isolates. Discrepancies among measured virulence in *Galleria mellonella* and the serum-killing assay, and genotypes and phenotypes were detected. The results illustrate the complexity and difficulty with the current knowledge of hypervirulence to predict the phenotype by using genetic and phenotypic markers. Additionally, the emergence of carbapenem resistance in two isolates of KPC-2-producing hvKP of different sequence types emphasizes the urgency with which reliable clinical diagnostics for hvKP is needed.

## Introduction

*Klebsiella pneumoniae* is an opportunistic, gram-negative pathogen that is frequently associated with various hospital-associated infections (Bengoechea and Sa Pessoa, 2019). *K. pneumoniae* resistant to last-resort antibiotics (e.g., carbapenems) are becoming increasingly common worldwide (Lee et al., 2016). Although *K. pneumoniae* infections mostly affect elderly and immunocompromised individuals, an increasing prevalence of hypervirulent strains causing infections among young and otherwise healthy individuals has been reported, particularly in Southeast Asia. Infections with these hypervirulent *K. pneumoniae* (hvKP) are frequently community acquired and the strains show the ability to metastasize to and cause infections at unusual sites and are known to cause severe conditions including pyogenic liver abscesses, endophthalmitis, and meningitis (Paczosa and Mecsas, 2016).

In contrast to classical *K. pneumoniae* which are frequently multidrug-resistant (MDR) and even carbapenem-resistant, hvKP strains have rarely been reported as MDR until the recent decade (Shon et al., 2013; Paczosa and Mecsas, 2016). Following sporadic reports of occasional clinical isolates displaying a convergence of the phenotypes, Gu et al. (2018) reported on a lethal outbreak at a Chinese hospital of a carbapenem-resistant and hypervirulent *K. pneumoniae* (CRhvKP) clone of ST11 that had acquired hypervirulence-associated factors encoded on a plasmid. ST11 is the most prevalent CRKP clone in China, and subsequent reports indicate that ST11 strains having acquired the hypervirulence phenotype has become the most prevalent CRhvKP population in China and an emerging high-risk clone (Lee et al., 2017; Zhou et al., 2020). Nonetheless, sporadic reports on CRhvKP of other STs are increasing in prevalence (Lee et al., 2017; Dong et al., 2018; Liu et al., 2019), indicating that the convergence of carbapenem resistance and hypervirulence in *K. pneumoniae* is not isolated to a single genetic event and that, worryingly, the number of CRhvKP strains is increasing.

In a previous study, we collected clinical isolates of *K. pneumoniae* screening-positive for carbapenem resistance from blood cultures of patients with bloodstream infections at a large tertiary hospital in Hangzhou, China, from January 2013 to June 2017 (Zhou et al., 2020). The isolates were screened for carriage of either or both *rmpA* and *rmpA2* with whole-genome sequencing (WGS). A total of 165 isolates were identified, and most (*n* = 154) were found to belong to ST11. The characterization of the CRhvKP ST11-lineage has been described elsewhere (Zhou et al., 2020). The aim of the current study was to characterize the 11 non-ST11 isolates in terms of antimicrobial drug resistance profiles, antibiotic resistance genes, and virulence, in order to evaluate the contribution of different combinations of virulence factors on pathogenicity among a genetically diverse set of putatively hypervirulent and antibiotic-resistant *K. pneumoniae*. In addition, the aim was to investigate the emergence of carbapenem resistance and MDR among different, non-ST11 hvKP strains or the emergence of the hypervirulence phenotype among carbapenem-resistant and MDR strains of *K. pneumoniae*.

## Materials and Methods

### Collection and Selection of Bacterial Isolates

The setting and sampling procedures in the study have been described in detail elsewhere (Zhou et al., 2020). Briefly, a retrospective collection of 705 nonrepetitive *K. pneumoniae* clinical isolates obtained from blood cultures from patients with bloodstream infections at a 2,500-bed tertiary care hospital in Eastern China was performed between January 2013 and June 2017. The isolates included in this study fulfilled the following criteria: screening-positive for carbapenem non-susceptibility, positive for carriage of either or both regulators of hypermucoid phenotype A genes *rmpA* and *rmpA2*, and belonged to STs other than ST11. Species determination was performed by using matrix-assisted laser desorption ionization time-of-flight (MALDI-TOF) mass spectrometry (Bruker Daltonics, Bremen, Germany). Isolates non-susceptible to carbapenems were screened for by using the VITEK-II system (bioMérieux, Marcy-l’Etoile, France). Isolates were considered carbapenem non-susceptible if the MICs of meropenem or imipenem were ≥2 mg/L in accordance with CLSI (2017).

### Whole-Genome Sequencing of Selected Isolates

Isolates found to be carbapenem non-susceptible and determined as *K. pneumoniae* were selected for WGS. Paired-end libraries (2 × 125 bp) were constructed from the isolates and were whole-genome sequenced on an Illumina HiSeq 2500 instrument (Illumina, San Diego, United States). Assembly of the sequence data was performed *de novo* after quality trimming (Qs ≥ 20) by using CLC Genomics Workbench v10.0 (QIAGEN, Hilden, Germany). Sequence reads generated by a PacBio RS II system (Pacific Biosciences, California, United States) was used for scaffolding with Unicycler v0.4.0. The RAST server^1^ was used for genome annotation. Antibiotic resistance genes and multilocus sequence type (MLST) were queried by using the databases at the Center for Genomic Epidemiology^2^. Querying of virulence factors and K-serotyping was performed by using Kleborate^3^. Non-ST11 isolates found to carry either or both of *rmpA* and *rmpA2* were selected for further analyses.

### Antimicrobial Susceptibility Testing

The antibiotic susceptibility profile of the non-ST11 isolates were further confirmed by using the broth microdilution method according to CLSI (2017). MICs were determined for amoxicillin, amoxicillin–clavulanic acid, piperacillin–tazobactam, cefazolin, cefepime, ceftriaxone, cefuroxime, ceftazidime, cefoxitin, moxalactam, aztreonam, imipenem, meropenem, gentamicin, amikacin, ciprofloxacin, levofloxacin, tigecycline, fosfomycin, polymyxin B, and trimethoprim–sulfamethoxazole. Susceptibility was interpreted according to clinical breakpoints from CLSI (2017). Isolates were considered MDR if non-susceptible to ≥1 agent in >3 antimicrobial categories (Magiorakos et al., 2012).

### Determination of Hypermucoviscous Phenotype

Carbapenem non-susceptible *K. pneumoniae* strains were tested for the hypermucoviscous phenotype by using two different methods: (i) The string test was performed as previously described (Shon et al., 2013). In short, isolates were cultivated on a sheep blood agar plate at 37°C overnight and the mucoviscosity of the overnight cultures was tested by streaking an inoculation loop through a colony. If a colony adhered to the inoculation loop and a string of >5 mm could be formed by pulling the inoculation loop upwards, the string test was deemed positive and the isolate was considered hypermucoviscous. (ii) The spin test was performed as described by Dorman et al. (2018). In brief, overnight cultures were centrifuged and resuspended in 1 × PBS. The suspension (600 μl) was applied to the top of a density gradient of various concentrations of Percoll (GE Healthcare) in 1 × PBS; 50, 35, and 15%, followed by centrifugation for 30 min at 3,000 g. *Escherichia coli* ATCC25922 was used as the negative control.

### Virulence Assessment With Serum Killing and *G. mellonella* Infection Assays

The survivability of the isolates when exposed to human serum was evaluated by using a serum killing assay. Blood was drawn from six healthy human volunteers who had signed written consent forms before participating in the study. Normal human serum was prepared fresh, and the inactivated serum was prepared by heating at 56°C for 30 min prior to use in assays. An overnight bacterial culture was diluted to a concentration of 5 × 10^8^ CFU/ml, and 20 μl of bacterial suspension was incubated with 180 μl of normal serum or inactivated serum at 37°C for 1 h. Samples were diluted and enumerated on Mueller–Hinton agar plates, and colonies were counted after overnight culture. The bacterial survival rate was calculated using the following formula: Bacterial survival rate = (number of colonies with normal serum/number of colonies with inactivated serum) × 100%. The experiment was performed in triplicate.

*K. pneumoniae* virulence was further characterized by using a *Galleria mellonella in vivo* infection assay. Overnight cultures of *K. pneumoniae* isolates were washed with PBS and adjusted to a concentration of 1 × 10^6^ CFU/ml. Ten *G. mellonella* larvae were injected with an inoculum of 1 × 10^6^ CFU, and the number of surviving larvae was recorded at every 8th hour up to 72 h. The experiment was performed in triplicate for each isolate. Control experiments were performed with *G. mellonella* larvae injected with an equal volume of PBS without bacteria. The survival rate of the *G. mellonella* larvae was calculated as the mean ratio of surviving larvae expressed in percentages.

## Results and Discussion

The biological basis for the hypervirulence phenotype in *K. pneumoniae* has not been clearly established and is likely enabled by a combination of virulence factors. For the isolates included in this study, we chose to evaluate the most commonly used markers for hvKP including hypermucoviscosity evaluation (Shon et al., 2013; Dorman et al., 2018), determination of hypervirulence-associated K-serotype loci (Bialek-Davenet et al., 2014), presence of genes encoding auxiliary siderophores (aerobactin, salmochelin, and yersiniabactin) (Paczosa and Mecsas, 2016), colibactin (Lu et al., 2017), and PEG344 (Bulger et al., 2017) and either or both the regulator of mucoid phenotype A genes *rmpA* and *rmpA2* (Paczosa and Mecsas, 2016), in addition to evaluating the virulence with serum-killing and *G. mellonella in vivo* survival assays.

**FIGURE 1:**
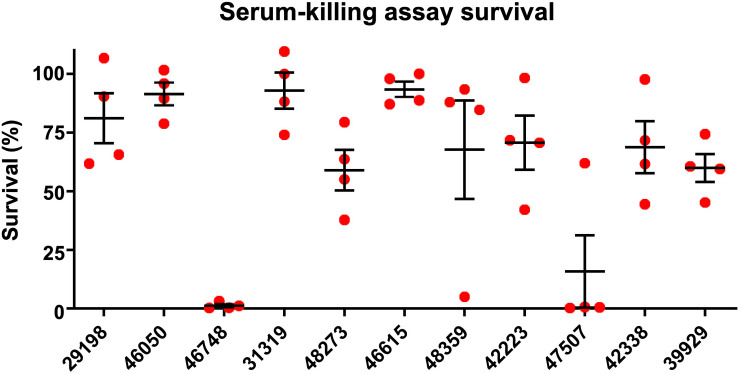
Serum-killing assay. Survival in a serum-killing assay of *K. pneumoniae* strains isolated from blood cultures of patients with bloodstream infections at a Chinese hospital. The survival is denoted in percentage. The bars denote means and standard errors of the mean.

**FIGURE 2:**
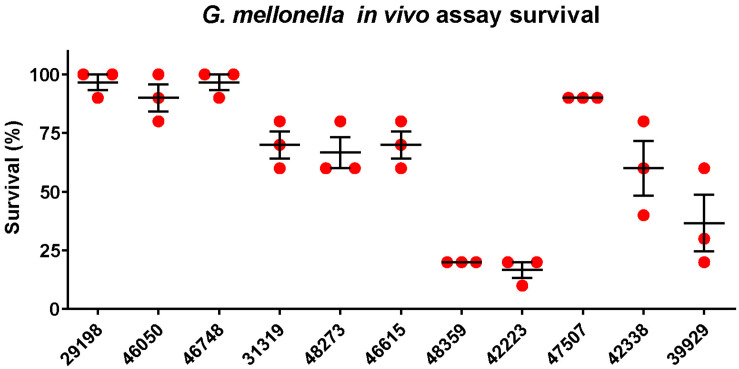
*Galleria mellonella* infection assay. Survival at 24 h in a *Galleria mellonella* assay of *K. pneumoniae* strains isolated from blood cultures of patients with bloodstream infections at a Chinese hospital. The survival is denoted in percentage. The bars denote means and standard errors of the mean.

A total of 11 *K. pneumoniae* isolates were selected and were characterized with WGS, MIC-determination, and virulence assays in the current study. Characteristics of the 11 isolates are summarized in Table 1. The isolates originated from patients with abdominal infections (*n* = 4), primary bloodstream infections (*n* = 4), knee infections (*n* = 1), liver abscesses (*n* = 1), and intracranial infections (*n* = 1). The isolates were found to belong to ST23 (*n* = 2), ST15 (*n* = 2), ST268 (*n* = 2), ST660 (*n* = 2), ST86 (*n* = 1), ST65 (*n* = 1), and ST1660 (*n* = 1) and carried K-loci KL1 (*n* = 3), KL2 (*n* = 2), KL16 (*n* = 2), KL20 (*n* = 2), and KL24 (*n* = 2). Hypermucoviscosity was observed (i.e., positive string test) for six isolates (55%). The serum survivability was ≥90% for three isolates and ranged from 59 to 81% for six isolates (Figure 1). Two isolates showed low serum survivability, 1.2 and 16%, respectively. The *G. mellonella* infection assay was evaluated at 24 h after infection as the results did not markedly differ for any isolate after 72 h (Figure 2). Two of the isolates caused a low survivability (17–20%). For five isolates, an intermediate survivability was observed after infection (37–70%) and for four isolates, a high survivability (90–97%) was observed. Aerobactin- and yersiniabactin-encoding genes were detected in all isolates. Genes encoding salmochelin, RmpA, and PEG344 were detected in seven isolates (64%). *rmpA2* was detected in all isolates, but the gene carried frameshift mutations in nine isolates (82%); only two isolates carried *rmpA2* with an intact reading frame (Supplementary Figure S1). Additionally, colibactin-encoding genes were carried by six isolates (64%).

**TABLE 1:** Infection sites, serotypes, STs, virulence assay characteristics, and virulence-associated genotypes for 11 isolates of *K. pneumoniae*.

Isolate	KP29198	KP46050	KP46748	KP31319	KP48273	KP46615	KP48359	KP42223	KP47507	KP42388	KP39929
Source of infection	Intracranial	Bloodstream	Liver abscess	Knee	Abdominal	Abdominal	Bloodstream	Abdominal	Bloodstream	Abdominal	Bloodstream
KPC-2	+	−	−	+	+	−	−	+	−	+	+
MDR	−	−	−	+	+	−	−	+	−	+	+
K-Locus	KL1	KL1	KL1	KL16	KL16	KL2	KL2	KL20	KL20	KL24	KL24
MLST	ST1660	ST23	ST23	ST660	ST660	ST86	ST65	ST268	ST268	ST15	ST15
String test	+	+	+	−	−	+	+	+	−	−	−
Spin test	33.4% ± 2.2%	55.6% ± 2.3%	58.4% ± 1.8%	20.6% ± 2.2%	20.8% ± 0.7%	52.7% ± 7.3%	40.4% ± 5.7%	18.6% ± 2.1%	22.5% ± 4.5%	19.5% ± 2.2%	20.1% ± 1.6%
Serum survival	81%	96%	1.2%	93%	59%	93%	68%	71%	16%	69%	60%
*G. mellonella* survival (24 h)	97%	90%	97%	70%	67%	70%	20%	17%	90%	60%	37%
Aerobactin	+	+	+	+	+	+	+	+	+	+	+
Salmochelin	+	+	+	−	−	+	+	+	+	−	−
Yersiniabactin	+	+	+	+	+	+	+	+	+	+	+
Colibactin	+	+	+	−	−	−	+	+	+	−	−
*rmpA*	+	+	+	−	−	+	+	+	+	−	−
*rmpA2*	FS*	+	FS	FS	FS	+	FS	FS	FS	FS	FS
*peg-344*	+	+	+	−	−	+	+	+	+	−	−

*FS indicates the presence the gene albeit with a frameshift mutation, likely engendering a non-functional protein.

The results of the antimicrobial susceptibility testing and antibiotic resistance gene survey are presented in Table 2. MICs for the tested antibiotics are presented in Supplementary Table S1. All 11 isolates included in the study were determined as carbapenem non-susceptible in the screening by using the VITEK-II system. However, the verification of carbapenem non-susceptibility with MIC-determination by using the broth microdilution method showed that five isolates were susceptible to both imipenem and meropenem. Six isolates were determined to be resistant to imipenem, all of which carried the carbapenemase gene *bla*_KPC–2_. Four of the imipenem-resistant isolates were additionally non-susceptible to meropenem; however, all six isolates showed elevated MICs of meropenem compared to the wild type (MICs > 0.125 mg/L), and these six isolates were thus considered carbapenem-resistant. A total of five isolates were determined to be MDR (KP31319, KP48273, KP42223, KP42388, and KP39929), all of which were also carbapenem-resistant.

**TABLE 2:** Antimicrobial susceptibility and antibiotic resistance genes of 11 isolates of *K. pneumoniae* isolated from blood cultures from patients with bloodstream infections at a Chinese hospital. Carbapenem resistance genes are underlined.

Isolate	Susceptible to:	Non-susceptible to:	Antibiotic resistance genes
KP29198	MEM, GEN, AMK, CIP, LEV, TGC, POL, SXT	AMX, AMC, TZP, KZ, FEP, CRO, CXM, CAZ, FOX, MOX, ATM, IMP, FOS	*bla*_KPC–2_, *bla*_CTX–M–3_, *bla*_SHV–36_, *aac(6’)*Ib-cr, *dfrA14*, *fosA*, *qnrS1*, *oqxA*, *oqxB*, *arr-3*
KP46050	AMC, TZP, FEP, CRO, CXM, CAZ, FOX, MOX, ATM, IMP, MEM, GEN, AMK, CIP, LEV, TGC, FOS, POL, SXT	AMX, KZ	*bla*_SHV–36_, *fosA*, *oqxA*, *oqxB*
KP46748	AMC, TZP, FEP, CRO, CXM, CAZ, FOX, MOX, ATM, IMP, MEM, GEN, AMK, CIP, LEV, TGC, FOS, POL, SXT	AMX, KZ	*bla*_SHV–36_, *fosA*, *oqxA*, *oqxB*
KP31319	FEP, FOX, MOX, GEN, AMK, TGC, POL, SXT	AMX, AMC, TZP, KZ, CRO, CXM, CAZ, ATM, IMP, MEM, CIP, LEV, FOS	*bla*_KPC–2_, *bla*_SHV–1_, *dfrA14*, *fosA*, *qnrS1*, *oqxA*, *oqxB*
KP48273	GEN, AMK, FOS, POL,	AMX, AMC, TZP, KZ, FEP, CRO, CXM, CAZ, FOX, MOX, ATM, IMP, MEM, CIP, LEV, TGC, SXT	*bla*_KPC–2_, *bla*_SHV–1_, *dfrA14*, *fosA*, *qnrS1*, *oqxA*, *oqxB*
KP46615	AMC, TZP, KZ, FEP, CRO, CXM, CAZ, FOX, MOX, ATM, IMP, MEM, GEN, AMK, CIP, LEV, TGC, FOS, POL	AMX, SXT	*bla*_SHV–1_, *fosA*, *oqxA*, *oqxB*
KP48359	AMC, TZP, KZ, FEP, CRO, CXM, CAZ, FOX, MOX, ATM, IMP, MEM, GEN, AMK, CIP, LEV, TGC, FOS, POL, SXT	AMX	*bla*_SHV–11_, *fosA*, *oqxA*
KP42223	MOX, MEM, GEN, AMK, CIP, LEV, TGC, FOS, POL, SXT	AMX, AMC, TZP, KZ, FEP, CRO, CXM, CAZ, FOX, ATM, IMP	*bla*_KPC–2_, *bla*_SHV–11_, *fosA*, *oqxA*, *oqxB*
KP47507	AMC, TZP, KZ, FEP, CRO, CXM, CAZ, FOX, MOX, ATM, IMP, MEM, GEN, AMK, CIP, LEV, POL, SXT	AMX, TGC, FOS	*bla*_SHV–11_, f*osA*, *oqxA*, *oqxB*
KP42388	TGC, POL	AMX, AMC, TZP, KZ, FEP, CRO, CXM, CAZ, FOX, MOX, ATM, IMP, MEM, GEN, AMK, CIP, LEV, FOS, SXT	*bla*_KPC–2_, *bla*_DHA–1_, *armA*, *sulI*, *mph(A)*, *msr(E)*
KP39929	GEN, AMK, TGC, POL	AMX, AMC, TZP, KZ, FEP, CRO, CXM, CAZ, FOX, MOX, ATM, IMP, MEM, CIP, LEV, FOS, SXT	*bla*_KPC–2_

*AMX: amoxicillin; AMC: amoxicillin-clavulanic acid; TZP: piperacillin-tazobactam; KZ: cefazolin; FEP: cefepime; CRO: ceftriaxone; CXM: cefuroxime; CAZ: ceftazidime; FOX: cefoxitin; MOX: moxalactam; ATM: aztreonam; IMP: imipenem; MEM: meropenem; GEN: gentamicin; AMK: amikacin; CIP: ciprofloxacin; LEV: levofloxacin; TGC: tigecycline; FOS: fosfomycin; POL: polymyxin B; SXT: trimethoprim-sulfamethoxazole.*

Out of a total of 11 isolates analyzed, 7 isolates belonged to hypervirulence-associated K-serotypes; K1 (*n* = 3), K2 (*n* = 2), and K20 (*n* = 2). Two K1 isolates belonged to ST23 (KP46050 and KP46748) and one belonged to ST1660 (KP29198). All K1 isolates were hypermucoviscous and carried genes encoding aerobactin, salmochelin, yersiniabactin, and colibactin, in addition to *rmpA* and *rmpA2* (although *rmpA2* was substantially truncated by a frameshift mutation in KP29198 and KP46748) (Supplementary Figure S1). They also carried the hypervirulence-associated gene *peg-344*. Although neither isolate was MDR, the ST1660 isolate (KP29198) carried *bla*_KPC–2_. Both KP46748 and KP29198 were isolated from patients with typical hvKP indications; liver abscess and intracranial infection, respectively. Interestingly, despite the clear hvKP clinical manifestations in the patients from which KP46748 and KP29198 originated, both isolates displayed very low virulence in the *G. mellonella in vivo* model (97% of larvae survived after 24 h). Whereas KP29198 showed fairly high serum survival (81%), KP46748 had very low serum survival (1.2%). Nonetheless, due to the clinical indications, KP46748 and KP29198 should be considered hypervirulent. It is noteworthy that the serum killing assay failed to predict high virulence in KP46748 and that the *in vivo* model failed to predict high virulence in both isolates.

The K2 isolates, KP46615 and KP48359, belonged to ST86 and ST65, respectively, and were both hypermucoviscous. Apart from KP46615, which did not carry genes encoding colibactin, these two isolates carried all the tested virulence genes. Although the ST86 isolate had higher serum survival compared to the ST65 isolate (93% as compared to 68%), the ST65 isolate showed a markedly higher virulence in the *G. mellonella* model (20% larvae survival as compared to 70% after 24 h). Neither of the isolates were carbapenem-resistant, nor were they MDR.

The two isolates of the hypervirulence-associated K20-serotype both belonged to ST268 (KP42223 and KP47507). KP42223 was MDR and carried *bla*_KPC–2_, whereas KP47507 was non-MDR and carbapenem susceptible. Both isolates carried genes encoding aerobactin, salmochelin, yersiniabactin, colibactin, and *peg-344*. Additionally, both isolates carried the same *rmpA* allele and a frameshifted *rmpA2*; however, in KP42223, the frameshift occurred late in the reading frame and only affected the 12 C-terminal amino acids (Supplementary Figure S1). KP42223 was hypermucoviscous whereas KP47507 was not, and the former showed fairly high serum survival (71%) and high virulence in the *G. mellonella* model (17% larvae survival after 24 h) whereas KP47507 had low serum survival (16%) and *G. mellonella* virulence (90% larvae survival after 24 h). These results indicate that while KP42223 is likely more virulent than KP47507 despite carrying the same siderophore operons and having identical K-serotype and *rmpA* allele. KP42223 was hypermucoviscous whereas KP47507 was not, which could be a significant contributor to virulence in the former, nonetheless, no explanation for the discrepancy in hypermucoviscous phenotype between the isolates could be found among the investigated genetic data.

In addition to KP42223, four isolates were both MDR and carried *bla*_KPC–2_, and belonged to ST15 (KP42388 and KP39929) and ST660 (KP31319 and KP48273), respectively. The ST15 isolate KP42388 additionally carried *armA*, a gene encoding a 16S rRNA methylase that engenders high-level aminoglycoside resistance and was the only isolate in the study resistant to the aminoglycosides gentamicin and amikacin. These four isolates were negative in the string test, and carried only aerobactin, yersiniabactin, and *rmpA2* among the tested virulence genes; however, *rmpA2* was likely not functional due to the presence of frameshift mutations substantially truncating the gene in all four isolates (Supplementary Figure S1). The serum survival in the isolates ranged from high in KP31319 (93%) to intermediate (59–69%) in the other isolates. The virulence in the *G. mellonella* model was also moderate for all four isolates (37–70% larvae survival after 24 h). The four isolates did not carry hypervirulence-associated K-loci. The scarcity of genetic virulence markers and the moderate virulence displayed in the virulence assays are indicative of non-hvKP strains. Indeed, the carbapenem resistance and MDR phenotypes are also consistent with classical *K. pneumoniae* strains causing hospital-associated infections, making it perhaps likelier that these isolates are not hypervirulent. The four isolates were the only isolates in the study to show resistance to fluoroquinolones (ciprofloxacin and levofloxacin). Fluoroquinolone resistance has been indicated as a major facilitating factor in propagating the clonal expansion of major STs (e.g., ST15), particularly via low-fitness cost fluoroquinolone resistance-yielding point mutations in chromosomal genes (Tóth et al., 2014; Fuzi et al., 2017). It has been observed that strains of minor STs incapable of developing energetically favorable fluoroquinolone resistance mutations have a tendency to forego acquisition of more fitness costly resistance mechanisms, and remain fluoroquinolone susceptibility, but are on the other hand more capable of acquiring an extensive load of virulence genes (Fuzi et al., 2020). These observations are consistent with the results of the current study.

Capsule overproduction, engendering the hypermucoviscous phenotype, has been strongly associated with hvKP. In this study, six isolates were determined as hypermucoviscous with the string test, all of which carried hypervirulence-associated K-loci (K1, K2, or K20), two of which are likely hypervirulent. The string test has been proposed as a screening method for hvKP; however, whereas the hypermucoviscosity and hypervirulence seem to be associated, the hypermucoviscous phenotype appears to be neither sufficient nor a prerequisite for hypervirulence; thus, the reliability of the string test for the identification of hvKP remains controversial (Catalán-Nájera et al., 2017; Russo et al., 2018). In addition to string test, we also evaluated the hypermucoviscous phenotype by using the spin test (Dorman et al., 2018). The results were fairly consistent with the string test (Supplementary Figure S2), and all isolates >30% in the spin test were also positive in the string test (except for KP42223) (Table 1).

The genes *rmpA* and *rmpA2* are regulatory genes that have been identified to be strongly associated with the hypermucoviscous phenotype. Either or both of these genes are commonly detected among hvKP (Paczosa and Mecsas, 2016); however, both genes contain several sections of single-nucleotide repeats that frequently are the target of indel mutations causing frameshifts, likely rendering the gene product dysfunctional (Hsu et al., 2011; Struve et al., 2015). In the current study, in which carriage of either gene was an inclusion criterion, all 11 isolates were found to carry *rmpA2*; however, 9 of those isolates had *rmpA2* alleles frameshifted by mutations, likely hampering the functionality of the gene product (Supplementary Figure S1). For this reason, screening for these genes may need to be followed up by sequencing to ensure gene integrity.

Certain virulence genes and operons are overrepresented among hvKP and are likely key factors in enabling virulence in hypervirulent strains and facilitating their survival in different tissues. The most clearly established hypervirulence-associated operons are the aerobactin, yersiniabactin, and salmochelin operons encoding accessory siderophores, proteins that chelate and scavenge iron (Shon et al., 2013; Paczosa and Mecsas, 2016). These siderophores are overrepresented among hvKP; however, each siderophore’s contribution to hypervirulence is unclear (Paczosa and Mecsas, 2016). In the current study, all isolates carried aerobactin and yersiniabactin. Although salmochelin was less common, it was carried by seven isolates, all of which belonged to hypervirulence-associated K-serotypes.

Colibactin, a polyketide-peptide genotoxin, has been shown to be necessary but insufficient for causing meningitis in an *in vivo* mouse model (Lu et al., 2017), and PEG344, a metabolite transporter, has been shown to be highly associated with hvKP strains in addition to being necessary for full virulence in mice after pulmonary challenge (Bulger et al., 2017). Six isolates in the current study carried the colibactin operon, including an isolate from a patient with intracranial infection whereas *peg-344* was detected among seven isolates. Both virulence factors were only found among isolates of hypervirulence-associated K-serotypes.

The serum-killing assay and the *G. mellonella in vivo* infection model failed to predict hypervirulence for a liver abscess-associated isolate, and two identical isolates in terms of virulence genes tested displayed highly different levels of virulence in the virulence assays. Discrepancies between measured virulence of *K. pneumoniae* in *G. mellonella* and in humans have previously been reported (McLaughlin et al., 2014). A recent study similarly showed the failure of a *G. mellonella in vivo* model to accurately differentiate between hvKP and non-hvKP strains (Russo and MacDonald, 2020). In comparison, a murine infection model accurately predicted the virulence phenotype among the strains. However, the authors also noted that their hvKP-type strains possessed a high number of hvKP-associated virulence factors; the murine model’s ability to predict virulence in strains containing fewer genetic hypervirulence markers is therefore unclear.

Hypervirulence in *K. pneumoniae* is essentially defined by its clinical manifestations; however, reliable testable markers are currently lacking. Russo et al. (2018) evaluated a number of genotypic and phenotypic hypervirulence markers and showed that virulence genes including *rmpA*, *rmpA2*, *iucA* (belonging to the aerobactin operon), and the PEG344-encoding gene *peg-344* could accurately be used to differentiate between cohorts of hvKP and non-hvKP. However, in that study, the non-hvKP cohort consisted of isolates from low-endemic countries (i.e., England, Canada and the United States) and the predictive value of each virulence factor is likely to be lower among isolates from the Asian Pacific Rim area where sporadic carriage of hypervirulence-associated genes among non-hvKP may be considerably more common. The results from the current study show the complexity and difficulty with the current knowledge of hypervirulence to predict the phenotype by using genetic markers and phenotypic and virulence assays. Notably, in the current study, all isolates belonging to hypervirulence-associated serotypes carried all the tested virulence factor genes (except for KP46615, which was negative for colibactin), however, there were considerable discrepancies in performance of the isolates in the *in vivo* and *in vitro* virulence assays. Further analyzes of additional hvKP strains with diverse genetic backgrounds are needed to elucidate the mechanisms behind the discrepancies observed here. To be able to design reliable diagnostic methods, it is likely necessary to further elucidate the mechanisms behind hypervirulence. In addition to elucidating each virulence factor’s individual contribution to hypervirulence, synergy effects between different virulence factors also need to be established. Furthermore, there are likely important contributing factors to hypervirulence that remain to be discovered. Future studies should focus on elucidating these aspects. The results from this study further show the emergence of a K1 and a K20 CRhvKP carrying *bla*_KPC–2_ belonging to ST1660 and ST268, respectively. It is likely that the convergence of the hypervirulence and carbapenem resistance phenotypes will continue to emerge in dissipate strains of *K. pneumoniae*, which further increases the urgency by which reliable hypervirulence markers need to be determined, to enable the design of an efficient diagnostic method for rapid determination of the hypervirulence phenotype in *K. pneumoniae*.

## Data Availability Statement

The WGS datasets generated in this study have been deposited at DDBJ/ENA/GenBank under the accession numbers: WAQX00000000, WAQY00000000, WAQZ00000000, WARA00000000, WARB00000000, WARC00000000, WARD00000000, WARE00000000, WARF00000000, WARG00000000, and WARH00000000.

## Ethics Statement

This study was approved by the Institutional Review Board of the First Affiliated Hospital of Zhejiang University in China (No. 2017-442).

## Author Contributions

KZ, PS, BB, and YX participated in the design and/or discussion of the study and revised it for important intellectual improvement. KZ, PS, YC, YZ, and TX carried out the major experiments. KZ, PS, and BB analyzed the data. KZ and BB wrote the manuscript. All authors read and approved the final version to be published.

## Conflict of Interest

The authors declare that the research was conducted in the absence of any commercial or financial relationships that could be construed as a potential conflict of interest.

## Footnotes

1 https://rast.nmpdr.org

2 https://cge.cbs.dtu.dk

3 https://github.com/katholt/Kleborate

## Supplementary Material

FIGURE S1 *rmpA2* mutations. Predicted gene product of *rmpA2* of 11 isolates of *K. pneumoniae* isolated from blood cultures of patients with bloodstream infections at a Chinese hospital. Gene products from several of the isolates were predicted to be truncated by frameshift mutations.

FIGURE S2 Capsule expression estimated by Percoll gradient centrifugation. Hypermucoviscous isolates are indicated in red.

TABLE S1 MICs of 21 antibiotics for *K. pneumoniae* isolated from blood cultures from patients with bloodstream infections at a Chinese hospital.
