# Supplementary material for: Hypervirulence Markers Among Non-ST11 Strains of Carbapenem- and Multidrug-Resistant Klebsiella pneumoniae Isolated From Patients With Bloodstream Infections
Source: Front Microbiol. 2020 Jun 18;11:1199. doi: 10.3389/fmicb.2020.01199 (PMC7314899; doi:10.3389/fmicb.2020.01199)
Supplement: FIGURE S1 — rmpA2 mutations. Predicted gene product of rmpA2 of 11 isolates of K. pneumoniae isolated from blood cultures of patients with bloodstream infections at a Chinese hospital. Gene products from several of the isolates were predicted to be truncated by frameshift mutations. [file Data_Sheet_1.PDF]

**KP46615**	MEKYIYFICNKDVNIVLTDDYFFYYGLKQLTGLPLFHITYEGVVNKSIAIKHKRNIRVLVDSRIFYSGKWGGYKMLRGSLNMISQWMWLDVSGGGRFYPKGCDYDIYVNMQGNVKNNIEKLYFAFLKKNVSR IVNHYPRLTKKEQAVLQCLLKNGGINEIKSQLKIEEKTLSCYQSKITRKFGCKRYIRFMYLYSLNKEMVDERWLMPSI
**KP47507**	MEKYIYFICNKDVNIVLTDDYFFYYGLKQLTGLPLFHITYEGVVNKSIAIKHKRNIRVLVDSRIFYSGKWGGYKMLRGSLNMISQWMWLDVSGGGGFYPKGCDYDIYVNMQGNVKNNIEKLYFAFLKKNVSR IVNHYPRLTKKEQAVLQCLLKNGGINEIKSQLKIEEKTLSCYQSKITRKFGCKRYIRFMYLYSLNKEMVDERWLMPSI
**KP48359**	MEKYIYFICNKDVNIVLTDDYFFYYGLKQLTGLPLFHITYEGVVNKSIAIKHKRNIRVLVDSRIFYSGKWGGYKMLRGSLNMISQWMWLDVSGGGRFYPKGCDYDIYVNMQGNVKNNIEKLYFAFLKKNVSR IVNHYPRLTKKEQAVLQCLLKNGGINEIKSQLKIEEKTLSCYQSKITRKFGCKRYIRFMYLYSLNKEMVDERWLMPSI
**KP42223**	MEKYIYFMCNKDVTLVLTDDYYFYFGLKQLTGLPLVYITYEGSMDKPIVIKQKRNIRVLVDSRIFYSGKWDGYKMLRKTLNMISQWMWLDISGGEKFYPKGCDYDIYVNMQGNLKKNIEELYYAYLKKNVSR IGNHYPQLTKKEQIILQCLLSRREGIHELKSRLKIEEKTLSCHRCKITRKFGCKRFIRFMYLYNLNKEITDEKWCTSNT
**KP39929**	MEKYIYFMCNKDVTLVLTDDYYFYFGLKQLTGLPLVYITYEGSMDKPIVIKQKRNIRVLVDSRIFYSGKWDGYKMLRKTLNMISQWMWLDISGGEKFYPKGCDYDIYVNMQGNLKKTLKSYIMHT
**KP42338**	MEKYIYFMCNKDVTLVLTDDYYFYFGLKQLTGLPLVYITYEGSMDKPIVIKQKRNIRVLVDSRIFYSGKWDGYKMLRKTLNMISQWMWLDISGGEKFYPKGCDYDIYVNMQGNLKKTLKSYIMHT
**KP29198**	MEKYIYFMCNKDVTLVLTDDYYFYFGLKQLTGLPLVYITYEGSMDKPIVIKQKRNIRVLVDSRIFYSGKWDGYKMLRKTLNMISQWMWLDISGGGGSFILKGVIMTSMSTCKEI
**KP46050**	MEKYIYFMCNKDVTLVLTDDYYFYFGLKQLTGLPLVYITYEGSMDKPIVIKQKRNIRVLVDSRIFYSGKWDGYKMLRKTLNMISQWMWLDISGGGRSFILKGVIMTSMSTCKEI
**KP31319**	MEKYIYFMCNKDVTLVLTDDYYFYFGLKQLTGLPLVYITYEGSMDKPIVIKQKRNIRVLVDSRIFYSGKWDGYKMLRKTLNMISQWMWLDISGGGGRSFILKGVIMTSMSTCKEI
**KP46748**	MEKYIYFMCNKDVTLVLTDDYYFYFGLKQLTGLPLVYITYEGSMDKPIVIKQKRNIRVLVDSRIFYSGKWDGYKMLRKTLNMISQWMWLDISGGGEVLS
**KP48273**	MEKYIYFMCNKDVTLVLTDDYYFYFGLKQLTGLPLVYITYEGSMDKPIVIKQKRNIRVLVDSRIFYSGKWDGYKMLRKTLNMISQWMWLDISGGGGEVLS
