# Supplementary figures and images for: Hypervirulence Markers Among Non-ST11 Strains of Carbapenem- and Multidrug-Resistant Klebsiella pneumoniae Isolated From Patients With Bloodstream Infections
Source: Front Microbiol. 2020 Jun 18;11:1199. doi: 10.3389/fmicb.2020.01199 (PMC7314899; doi:10.3389/fmicb.2020.01199)

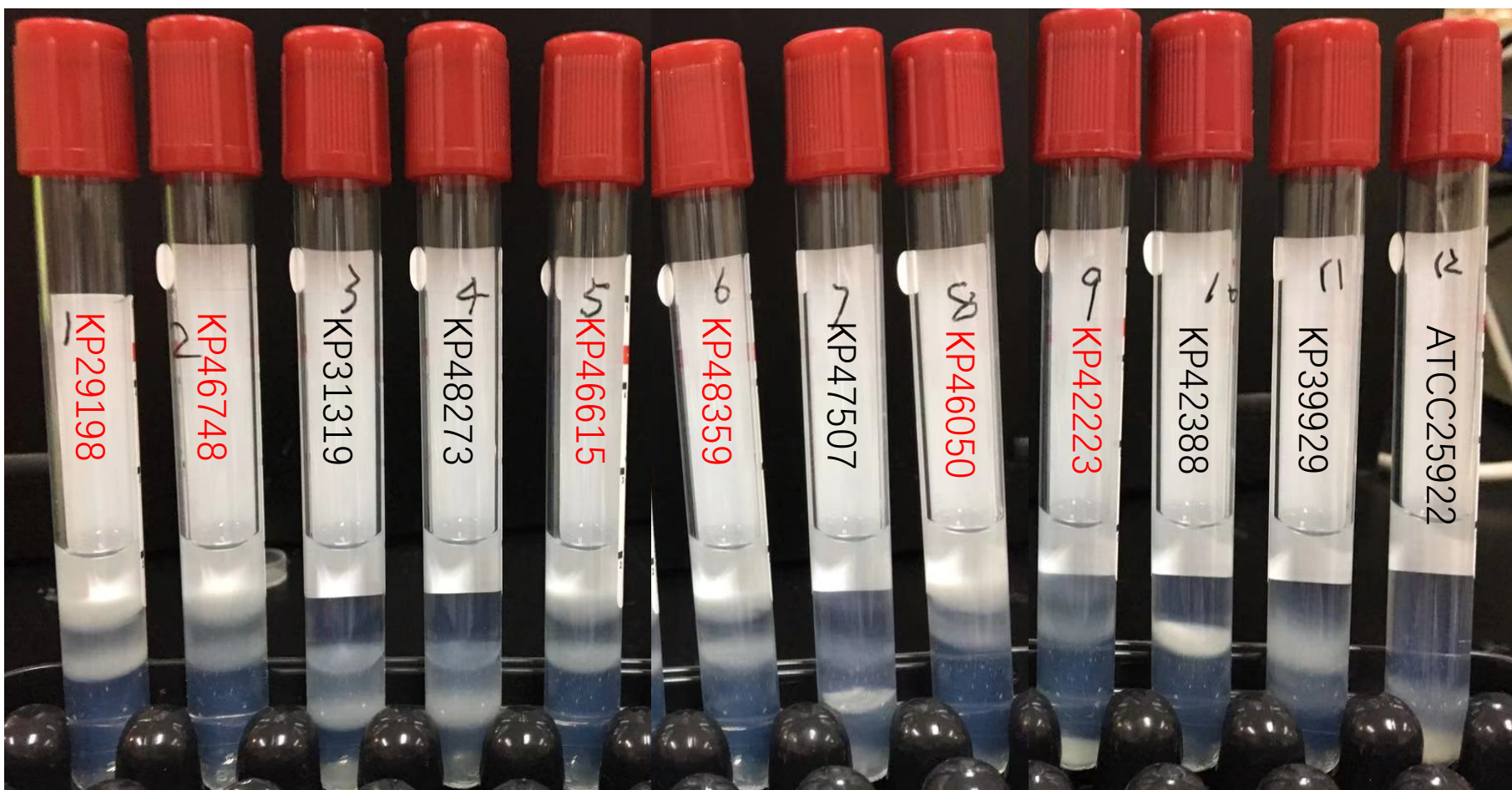

Supplement: FIGURE S2 — Capsule expression estimated by Percoll gradient centrifugation. Hypermucoviscous isolates are indicated in red. [file Data_Sheet_2.PDF]
