# Supplementary material for: Hypervirulence Markers Among Non-ST11 Strains of Carbapenem- and Multidrug-Resistant Klebsiella pneumoniae Isolated From Patients With Bloodstream Infections
Source: Front Microbiol. 2020 Jun 18;11:1199. doi: 10.3389/fmicb.2020.01199 (PMC7314899; doi:10.3389/fmicb.2020.01199)
Supplement: TABLE S1 — MICs of 21 antibiotics for K. pneumoniae isolated from blood cultures from patients with bloodstream infections at a Chinese hospital. [file Table_1.docx]

**Table S1.** MICs of 21 antibiotics for *K. pneumoniae* isolated from blood cultures from patients with bloodstream infections at a Chinese hospital.

|  | **KP29198** | **KP46050** | **KP46748** | **KP31319** | **KP48273** | **KP46615** | **KP48359** | **KP42223** | **KP47507** | **KP42338** | **KP39929** |
| --- | --- | --- | --- | --- | --- | --- | --- | --- | --- | --- | --- |
| **AMX** | >256 | 256 | >256 | >256 | >256 | 256 | 256 | >256 | 256 | >256 | >256 |
| **AMC** | >128/64 | 1/0.5 | 1/0.5 | 128/64 | >128/64 | 1/0.5 | 1/0.5 | 128/64 | 1/0.5 | >128/64 | >128/64 |
| **TZP** | 128/4 | 8/4 | 16/4 | >128/4 | >128/4 | 16/4 | 8/4 | >128/64 | 2/4 | >128/64 | >128/64 |
| **KZ** | >128 | 4 | 4 | >128 | >128 | 2 | 2 | >128 | 2 | >128 | >128 |
| **FEP** | 16 | 0.06 | 0.06 | 2 | >32 | 0.06 | 0.06 | 8 | 0.6 | >32 | >32 |
| **CRO** | >128 | 0.125 | 0.125 | 16 | >32 | 0.06 | 0.125 | 32 | 0.06 | >32 | >32 |
| **CXM** | >128 | 4 | 2 | >128 | 128 | 2 | 2 | >64 | 2 | >64 | >64 |
| **CAZ** | 64 | 0.25 | 0.25 | 8 | >64 | 0.25 | 0.25 | 16 | 0.25 | 64 | >64 |
| **FOX** | 16 | 8 | 8 | 8 | >128 | 4 | 8 | 16 | 4 | >128 | >128 |
| **MOX** | 8 | 0.25 | 0.5 | 4 | >64 | 0.25 | 0.25 | 8 | 0.25 | >128 | >128 |
| **ATM** | 128 | 0.06 | 0.06 | 128 | >64 | 0.06 | 0.06 | >64 | 0.06 | >64 | >64 |
| **IMP** | 4 | 0.25 | 0.25 | 4 | 32 | 0.25 | 0.5 | 4 | 0.25 | >32 | >32 |
| **MEM** | 1 | 0.06 | 0.125 | 2 | 8 | 0.06 | 0.06 | 1 | 0.125 | >32 | 32 |
| **GEN** | 1 | 1 | 2 | 0.25 | 1 | 1 | 1 | 1 | 1 | >128 | 1 |
| **AMK** | 4 | 2 | 8 | 0.5 | 1 | 2 | 2 | 1 | 2 | >128 | 1 |
| **CIP** | 1 | 0.06 | 0.06 | 16 | 16 | 0.06 | 0.125 | 0.06 | 0.06 | >32 | >32 |
| **LEV** | 1 | 0.06 | 0.06 | 8 | 16 | 0.06 | 0.125 | 0.06 | 0.06 | 32 | 32 |
| **TGC** | 0.5 | 2 | 1 | 2 | 4 | 1 | 2 | 1 | 4 | 2 | 2 |
| **FOS** | >128 | 16/25 | 32/25 | 64/25 | 2/25 | 4/25 | 16/25 | 16/25 | 64/25 | 128/25 | 128/25 |
| **POL** | 0.125 | 1 | 1 | 1 | 1 | 1 | 1 | 1 | 1 | 1 | 1 |
| **SXT** | 0.5/4.5 | 0.25/4.8 | 0.25/4.8 | 2/38 | >8/152 | >8/152 | 0.5/9.5 | 0.25/4.8 | 0.125/2.4 | >8/152 | 8/152 |

AMX: amoxicillin; AMC: amoxicillin-clavulanic acid; TZP: piperacillin-tazobactam; KZ: cefazolin; FEP: cefepime; CRO: ceftriaxone; CXM: cefuroxime; CAZ: ceftazidime; FOX: cefoxitin; MOX: moxalactam; ATM: aztreonam; IMP: imipenem; MEM: meropenem; GEN: gentamicin; AMK: amikacin; CIP: ciprofloxacin; LEV: levofloxacin; TGC: tigecycline; FOS: fosfomycin; POL: polymyxin B; SXT: trimethoprim-sulfamethoxazole
